# Isolation and molecular characterization of the indigenous *Staphylococcus aureus* strain K1 with the ability to reduce hexavalent chromium for its application in bioremediation of metal-contaminated sites

**DOI:** 10.7717/peerj.7726

**Published:** 2019-10-11

**Authors:** Muhammad Tariq, Muhammad Waseem, Muhammad Hidayat Rasool, Muhammad Asif Zahoor, Irshad Hussain

**Affiliations:** 1 Department of Microbiology, Government College University, Faisalabad, Punjab, Pakistan; 2 Department of Chemistry and Chemical Engineering, The Lahore University of Management Sciences (LUMS), Lahore, Punjab, Pakistan

**Keywords:** Chromium, Effluents, *Staphylococcus*, Environmental pollution, Metal tolerance

## Abstract

**Background:**

Urbanization and industrialization are the main anthropogenic activities that are adding toxic heavy metals to the environment. Among these, chromium (in hexavalent: Cr^+6^ and/or trivalent Cr^+3^) is being released abundantly in wastewater due to its uses in different industrial processes. It becomes highly mutagenic and carcinogenic once it enters the cell through sulfate uptake pathways after interacting with cellular proteins and nucleic acids. However, Cr^+6^ can be bio-converted into more stable, less toxic and insoluble trivalent chromium using microbes. Hence in this study, we have made efforts to utilize chromium tolerant bacteria for bio-reduction of Cr^+6^ to Cr^+3^.

**Methods:**

Bacterial isolate, K1, from metal contaminated industrial effluent from Kala Shah Kaku-Lahore Pakistan, which tolerated up to 22 mM of Cr^6+^ was evaluated for chromate reduction. It was further characterized biochemically and molecularly by VITEK^®^2 system and 16S rRNA gene sequencing respectively. Other factors affecting the reduction of chromium such as initial chromate ion concentration, pH, temperature, contact-time were also investigated. The role of cellular surface in sorption of Cr^6+^ ion was analyzed by FTIR spectroscopy.

**Results:**

Both biochemical and phylogenetic analyses confirmed that strain K1 was *Staphylococcusaureus* that could reduce 99% of Cr^6+^ in 24 hours at 35 °C (pH = 8.0; initial Cr^6+^ concentration = 100 mg/L). FTIR results assumed that carboxyl, amino and phosphate groups of cell wall were involved in complexation with chromium. Our results suggested that *Staphylococcusaureus* K1 could be a promising gram-positive bacterium that might be utilized to remove chromium from metal polluted environments.

## Introduction

Chromium is of great economic importance for its uses in different industries for various applications such as textile dyeing, wood preservation, pulp and paper manufacturing, tanning and chrome plating (Viti & Giovannetti, 2007). However, it is also becoming a major pollutant due to its untreated and uncontrolled discharge in industrial effluent in developed as well as in developing countries like Pakistan (Velma, Vutukuru & Tchounwou, 2009; Khan et al., 2015; Fatima & Ahmed, 2018). According to estimates, more than 170 thousand tons per annum of chromium waste are being discharged globally into environments due to various anthropogenic activities, thus causing severe environmental pollution and human health problems. In aquatic environment, chromium is found either chromium (III) or chromium (VI) (Kaur, Kumar & Kaur, 2014). Trivalent chromium (Cr^+3^) is least toxic as it got precipitated whereas chromate (Cr^+6^) is the most toxic form of chromium being released into environment by industrial effluents because of its strong oxidizing power and higher membrane transport (Viti et al., 2014). Chromate is actively transported across cell membranes both in prokaryotes and eukaryotes (Cervantes et al., 2001). Sulfate uptake pathways were reported to be involved in transport of chromate across biological membranes (Ramírez-Díaz et al., 2008) in bacteria such as *Salmonella typhimurium, Escherichia coli, Pseudomonas fluorescens* and *Alcaligenes eutrophus* (Cervantes et al., 2001). This is due to the fact that chromate is mainly found in the oxyanion form (i.e., CrO_2_Q) and, thus cannot be trapped by the anionic components of cell membrane (Volesky & Holan, 1995). Once it enters the cell, it may cause asthma, cancer, allergic reactions, nervous and cardiovascular disorders and organ failure (Gupta & Kumar, 2012). According to different environmental protection agencies, removal of chromium from industrial discharge is pre-requisite before its final dispersal to surface water bodies (Viti et al., 2014).

The proper treatment of wastewater effluent not only fulfills the demands for agricultural irrigation but also minimizes the environmental pollution and protects the public health (Al-Jasser, 2011). However, industrial wastewater treatment is a well-developed engineering science requiring several techniques and processes to efficiently treat the wastewater (Asano & Levine, 1996). Like other developing countries, hardly a few percent (<8%) of wastewater is effectively treated in Pakistan (Martin et al., 2006), while rest is directly discharged into surface water bodies without undergoing any sort of treatment (Reinsch & Pearce, 2005).

In developed countries, various physiochemical processes such as filtration, electrochemical application, ion exchange, evaporation, chemical precipitation, oxidation, reduction, and reverse osmosis, have been used for removal of toxic metals from environment (Hassan et al., 2017). These methods are not only expensive but also release other harmful byproducts leading to environment pollution (Ahluwalia & Goyal, 2007). On the other hand, bioremediation is gaining attention as an alternative remediation technique due to its inexpensive and environment friendly nature (Kothe, Bergmann & Büchel, 2005), and, also save the extra cost of secondary pollution removal.

Bioremediation of wastewater using metal resistant bacteria could be considered as cost-effective and environmentally benign approach. This is due to the premise that these bacteria are in continuous contact with wastewater containing toxic metals since years; therefore, they have already adapted these extreme environmental conditions. Additionally, these extreme environments could provide novel bacteria that could be resistant to elevated concentrations of toxic metals. These bacteria could be used for bioremediation of contaminated wastewater being discharged into surface water bodies. Therefore, this study was carried out to investigate the presence of chromium tolerant bacteria in industrial effluent for its subsequent applications in environmental remediation practices.

## Materials & Methods

### Collection of wastewater samples

**Figure 1:**
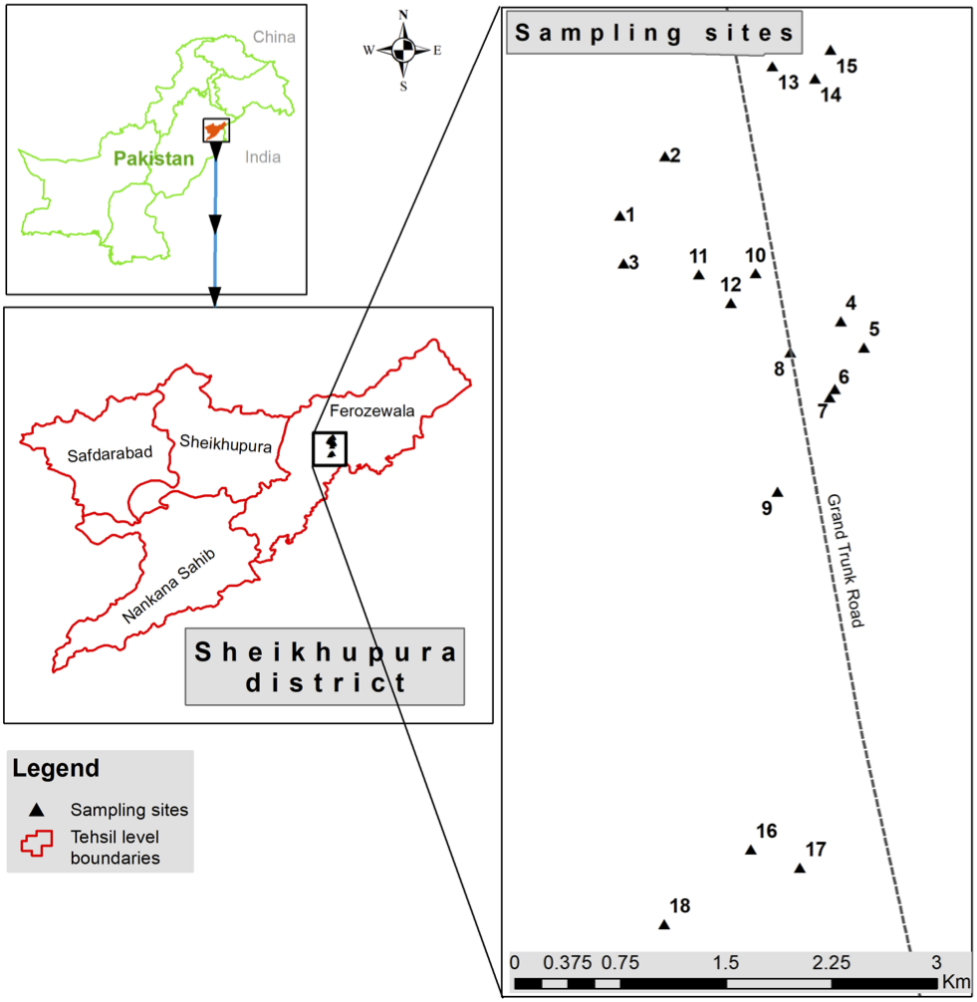
Sampling sites located at discharge points of different industries at Kala Shah Kaku, Lahore-Pakistan. This picture is based on GPS coordinates of collection points.

Wastewater samples (*n* = 6) were collected from discharge points of different industries located in industrial state of Kala Shah Kaku (KSK) near Lahore city, Pakistan (Fig. 1). Sampling bottles (250 ml) were washed with dilute nitric acid, rinsed with de-ionized water and again washed three times with the wastewater as described previously (Zhang et al., 2013). These samples were stored in icebox (at 4 °C) and transported to laboratory for further analyses.

### Isolation of chromium resistant bacteria

Modified serial dilution method was used for isolation of metal resistant bacteria from collected wastewater samples (Lucious et al., 2013). Briefly distilled water was used to prepare dilutions (10^−1^, 10^−2^, 10^−3^ and 10^−4^) of wastewater samples (Lucious et al., 2013). Wastewater samples (100 µl) from test tubes of all dilution was poured onto petri plates containing 20 ml of Tryptic Soy Agar incorporated with 0.5 mM Cr^6+^ (Lucious et al., 2013). Morphologically different colonies observed on petri plates were purified and were used for studying their ability to tolerate elevated concentrations of Cr^6+^ by culturing them on petri dishes. These petri dishes were supplemented with increasing concentrations (0.0, 0.5, 2.0, 5.0, 10.0, 15.0, 18.0, 20, 22 and 23 mM) of chromium (Cr^6+^) (Zhang et al., 2013). Only the bacterium that was resistant to elevated concentration of chromium was selected for further studies (Khan et al., 2015; Zhenggang et al., 2018).

### Minimum inhibitory concentration of different metals against bacterial isolate K1

The bacterial isolate, that was resistant to elevated concentration of hexavalent chromium (i.e., 22 mM), was purified and was tested for resistance against different metals like chromium, lead, copper, zinc, manganese, cobalt, silver and mercury by the agar dilution method (Khan et al., 2015). Briefly Tryptic Soy Agar (TSA) was prepared and respective metals were mixed with agar in various concentrations (i.e., 0.5, 1.0, 2.0, 4.0, 8.0, 10.0, 12.0, 15.0, 20.0, 22.0 and 23.0 mM). Then the plates were allowed to solidify. Now bacterial culture was streaked on the surface of TSA plates that were incubated at 35 °C for a period of 5 to 6 days and observed for growth on daily basis (Khan et al., 2015).

### Identification and biochemical characterization of chromium resistant bacteria

The bacterial isolate (K1) was identified on the basis of cell morphology and biochemical tests (Begum et al., 2017). Biochemical tests were performed using VITEK^®^2 system (bioMérieux, France) through colorimetric identification. This system uses GP card containing 43 different carbon sources, enzyme activities for fast and accurate microbial identification. The test was carried out for 8 h following the manufacturer instructions in which tested bacterium K1 was tested and compared with control strain i.e., *S. aureus* ATCC 6538.

### Molecular characterization and phylogenetic analysis of chromium resistant strain K1

For molecular characterization, genomic DNA was extracted by the Favorgen^®^ genomic DNA extraction kit following the manufacturer’s guidelines. 16S rDNA gene was amplified using universal primers set i.e., 27F (5′-AAACTCAAATGAATTGACGG-3′) & 1492R (5′-ACGGGCGGTGTGTAC-3′) through polymerase chain reaction (PCR) (Eden et al., 1991). The PCR was performed with initial denaturation temperature of 94 °C for 5 min, followed by 40 recurring cycles having denaturation of 30 s at 94 °C, 30 s of annealing at 53 °C and elongation for 30 s at 72 °C. The final extension time was 10 min at 72 °C followed by 4 °C hold temperature (Moyersoen, Beever & Martin, 2003). After this, PCR amplicon were visualized under ultra-violet light via Gel Documentation System (Slite 200W), after loading the 5 µl of PCR product on the agarose gels as described previously (Moyersoen, Beever & Martin, 2003). After confirmation, 25 µl of PCR product was sent to Macrogen (Korea) for sequencing. The nucleotide sequences were corrected manually using ChormasPro (v1.7.1) software and were submitted to GenBank for an accession number. Through NCBI blast analysis, similar sequences were searched and downloaded for the construction of the phylogenetic tree using partial 16S rDNA gene sequences by the computer software MEGA (v7.0.) (Tamura et al., 2011).

### Determination of Antibiotic Susceptibility of Strain K1

Kirby-Bauer disc diffusion method was adopted to evaluate the antibiotic sensitivity pattern of *Staphylococcus aureus* K1 against selected antibiotics (CLSI, 2013). Commercially available antibiotic discs (Oxoid™ UK) were used in this study including ceftriaxone (30 µg), ampicillin (10 µg), amoxicillin (30 µg), meropenem (10 µg), imipenem (10 µg), sulphamethoxazole/trimethoprim (25 µg), nalidixic acid (30 µg), cefoxitin (30 µg), ciprofloxacin (5 µg), ofloxacin (5 µg), ceftazidime (30 µg), aztreonam (30 µg), gentamycin (10 µg), vancomycin (30 µg), ertrapenem (10 µg), amikacin (30 µg), piperacillin/tazobactam (110 µg) and cefepime (30 µg) (Begum et al., 2017). The tests were conducted following manufacturer instructions. The values obtained for diameter were compared with CLSI (Clinical and Laboratory Standards Institute) standards (CLSI, 2013) in order to confirm antibiotic resistance/ or sensitivity pattern of *Staphylococcus aureus* K1.

### Estimation of effect of contact time (hours) on reduction of chromium (Cr^+6^) by *Staphylococcus aureus* K1

Effect of contact time (hours) was determined using total 10 sets of conical flasks, each set comprised of 3 flasks (250 ml) containing Tryptic Soy Broth (100 ml). These flasks were inoculated with *Staphylococcus aureus* K1 (1 ml) and were incubated at optimum temperature (i.e., 35 °C). Overnight culture of *Staphylococcus aureus* K1 (1ml) was pipetted in 100 ml of Tryptic Soy Broth (TSB) supplemented with 1 mM of hexavalent chromium in 5 sets of three conical flasks (250 ml) while remaining 5 sets of flasks were taken as control (without chromium). These flasks (10 sets) were incubated in incubator at 35 °C for a period of 2, 4, 8, 16 and 24 h on orbital shaker (at 150 rpm) and harvesting was done accordingly. Breifly, bacterial cells were harvested through centrifugation at 8,000 rpm for 10 min and supernatant (100 µl) was added in 10 ml of distilled water in a test tube, followed by addition of 1 ml of diphenylcarbazide solution (that was prepared by dissolving 0.25 g diphenylcarbazide in 100 ml acetone) and one drop of H_3_PO_4_. The mixture was kept at room temperature for 10 min for color development. Reduction of chromium was measured at different time intervals by measuring optical density (540 nm) of extracted solution using spectrophotometer (Hach, USA) with 1, 5-diphenylcarbazide assay (Fulladosa et al., 2006; Thacker et al., 2006). For the estimation of chromium concentrations in extracted solution, standard curve was plotted using values of optical density obtained for Tryptic Soy Broth (100 ml) with different chromium concentrations (i.e., 0, 0.05, 0.1, 0.2, 0.4, 0.6, 0.8 and 1.0 mM) without bacterial cells.

### Fourier-transform infrared spectroscopy (FTIR) analysis

For FTIR analysis, bacterial samples were prepared according to Deokar et al. (2013). *Staphylococcus aureus* K1 was cultured on TSB with 1 mM or without chromium (control) and incubated for 24 h at 35 °C. After incubation period, both cultures were independently centrifuged at 8,000 rpm for 10 min. The obtained pallet was washed twice with 1% (W/V) saline solution having pH 6.5 and was further freeze-dried. Fourier transform infrared studies (FTIR) were monitored using FTIR spectrometer (Bruker, alpha) between wave number ranging from 4,000–500 cm^−1^.

## Results

### Biochemical, physiological and molecular identification of isolate K1

**Figure 2:**
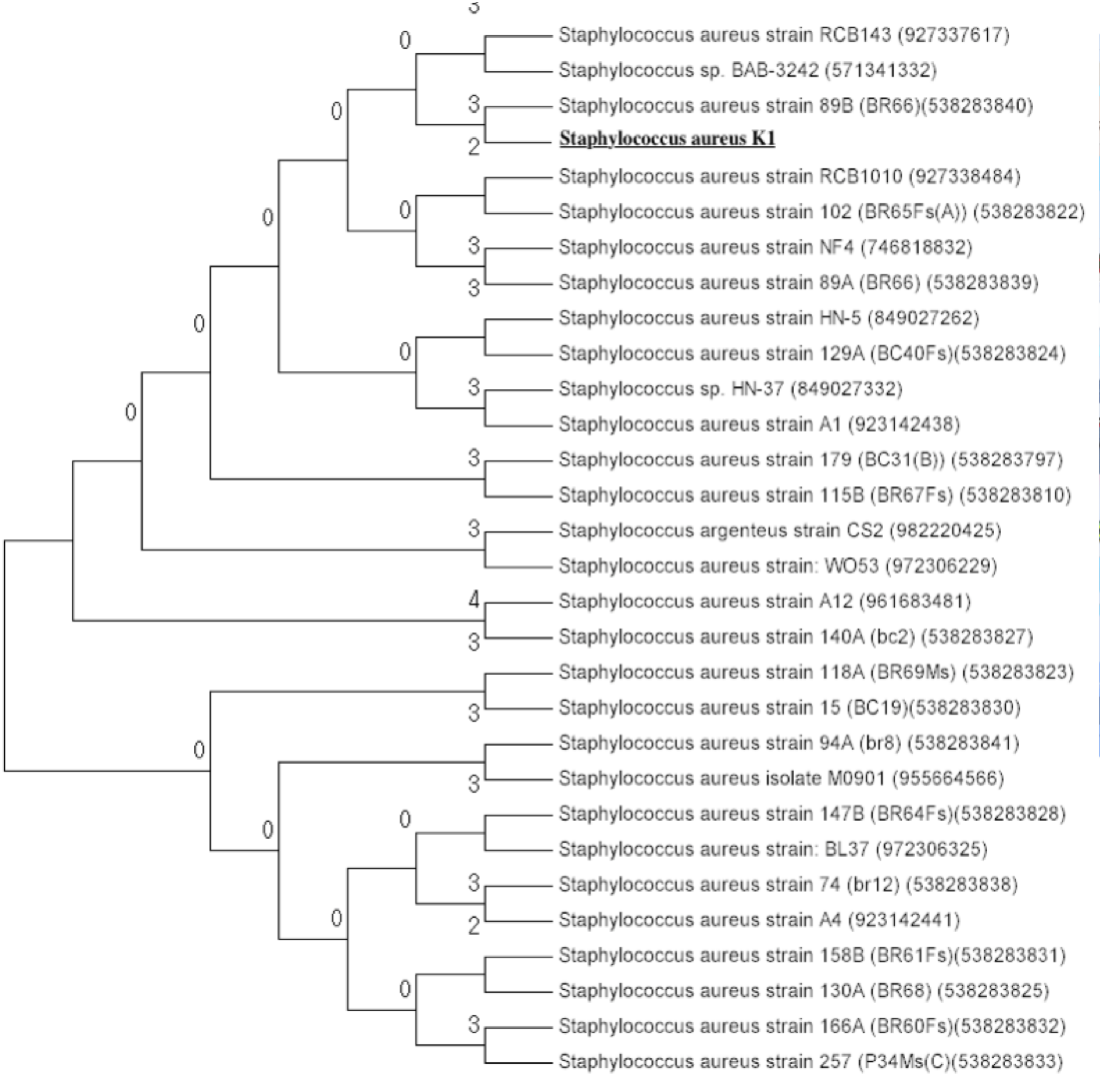
Phylogenetic tree of Staphylococcus aureus strain K1 isolated from industrial effluents based on their 16S rRNA gene sequences. The evolutionary trends of strain K1 are represented by Neighbor-Joining method (Saitou & Nei, 1987) are conducted with MEGA7 (Kumar, Stecher & Tamura, 2016). The evolutionary distance is estimated with the Tamura 3-parameter method (Tamura, 1992).

The isolate appeared to be coccus shaped (≈1 μm), gram-positive, catalase positive, oxidase and coagulase negative (Table 1). To further clarify the relationship between isolate K1 and *S. aureus*, BioMériux VITEK®2 system (GP card) was used for identification of isolate K1. Data obtained suggested that isolate K1 shared 37 out of 43 carbon sources with that of type strain *Staphylococcus aureus* ATCC 6538 whereas it differs in 6 carbon sources. Among them, bacterial isolate K1 used beta-galactosidase and lactose during growth, which were not used by *Staphylococcus aureus* ATCC 6538. On the other hand, *Staphylococcus aureus* ATCC 6538 assimilated α-glucosidase, D-maltose, and methyl-β-D-glucopyranoside as carbon sources for its growth that were not used by isolate K1. These results emphasized that bacterium K1, even after exhibiting significant differences from known strains, was closely related to *Staphylococcus aureus* and thus identified as *Staphylococcus aureus* strain K1. The BLASTn analysis applied to 16S rDNA gene sequence of *Staphylococcus aureus* K1 revealed that it was closely related (99% similarity) to *Staphylococcus aureus* strain NBRC 100910 and *Staphylococcus aureus* strain ATCC 12600. Phylogenetic analysis of 16S rDNA sequences that were retrieved from GenBank confirmed that isolate *Staphylococcus aureus* K1 appeared to be closely related to *Staphylococcus aureus* (Fig. 2).

**Table 1:** Comparative morphological and biochemical characteristics of Staphylococcus aureus strain K1 and Staphylococcus aureus ATCC strain 6538 using VITEK-2 Systems.

**Characters**	**Mnemonic**	*Staphylococcus aureus K1*	*Staphylococcus aureus ATCC 6538*
		**Morphological characteristics**	
Morphology		Round, Convex	Round, Convex
Color		Golden yellow	Golden yellow
Gram-reaction		Positive	Positive
Catalase		Positive	Positive
Coagulase plasma reaction		Negative	Positive
		**Biochemical characteristics**	
D-Amygdalin	AMY	−	−
Phosphatidylinositol phospholipase C	PIPLC	−	−
D-xylose	dXYL	−	−
Arginine dihydrolase I	ADH 1	+	+
Beta-Galactosidase	BGAL	+	−
Alpha-Glucosidase	AGLU	−	+
Ala-Phe-Pro Arylamidase	APPA	−	−
Cyclodextrin	CDEX	−	−
L-Aspartate Arylamidase	AspA	−	−
Beta-Galactopyranosidase	BGAR	−	−
Alpha-Mannosidase	AMAN	−	−
Phosphatase	PHOS	+	+
Leucine ARYLAMIDASE	LeuA	+	+
L-Proline ARYLAMIDASE	ProA	−	−
BETA GLUCURONIDASE	BGURr	−	−
ALPHA-GALACTOSIDASE	AGAL	−	−
L-Pyrrolidonyl-ARYLAMIDASE	PyrA	+	+
BETA-GLUCURONIDASE	BGUR	−	−
Alanine ARYLAMIDASE	AlaA	−	−
Tyrosine ARYLAMIDASE	TyrA	−	−
D-SORBITOL	dSOR	−	−
UREASE	URE	−	−
POLYMIXIN B RESISTANCE	POLYB	+	+
D-GALACTOSE	dGAL	+	+
D-RIBOSE	dRIB	−	−
L-LACTATE alkalinization	ILATk	+	+
LACTOSE	LAC	+	−
N-ACETYL-D-GLUCOSAMINE	NAG	+	+
D-MALTOSE	dMAL	−	+
BACITRACIN RESISTANCE	BACI	+	+
NOVOBIOCIN RESISTANCE	NOVO	−	+
GROWTH IN 6.5% Nacl	NC6.5	+	+
D-MANNITOL	dMAN	+	+
D-MANNOSE	dMNE	+	+
METHYL-B-D-GLUCOPYRANOSIDE	MBdG	−	+
PULLULAN	PUL	−	−
D-RAFFINOSE	dRAF	−	−
O/129 RESISTANCE (comp.vibrio)	O129R	+	+
SALICIN	SAL	−	−
SACCHAROSE/SUCROSE	SAC	+	+
D-TREHALOSE	dTRE	+	+
ARGININE DIHYDrolase 2	ADH2s	+	+
OPTOCHIN RESISTANCE	OPTO	+	+

### Determination of minimum inhibitory concentration for hexavalent chromium

**Figure 3:**
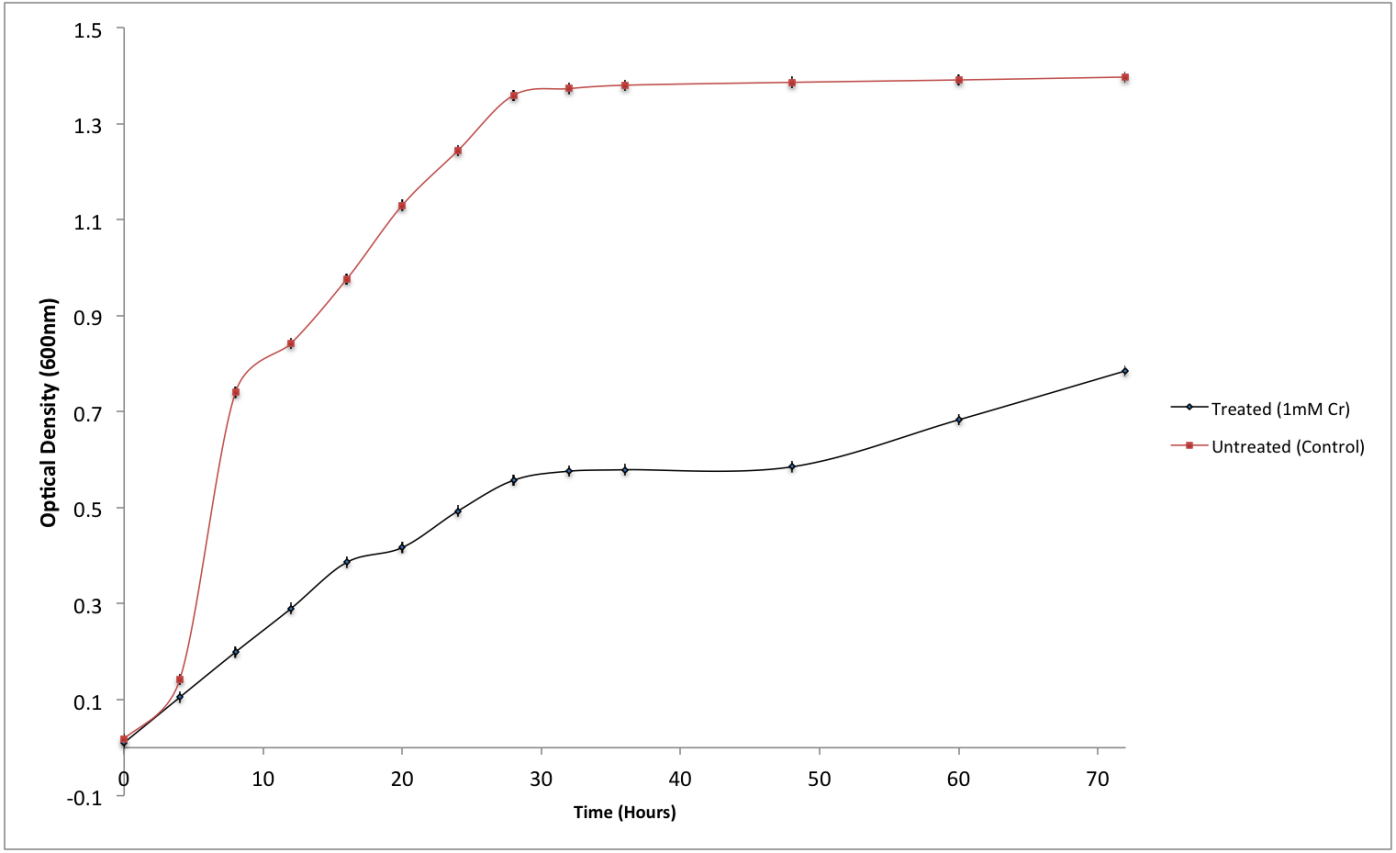
Effect of chromium (Cr^6+^) on the growth of *Staphylococcus aureus* strain K1. Each data point indicates the average performance of triplicates.

**Figure 4:**
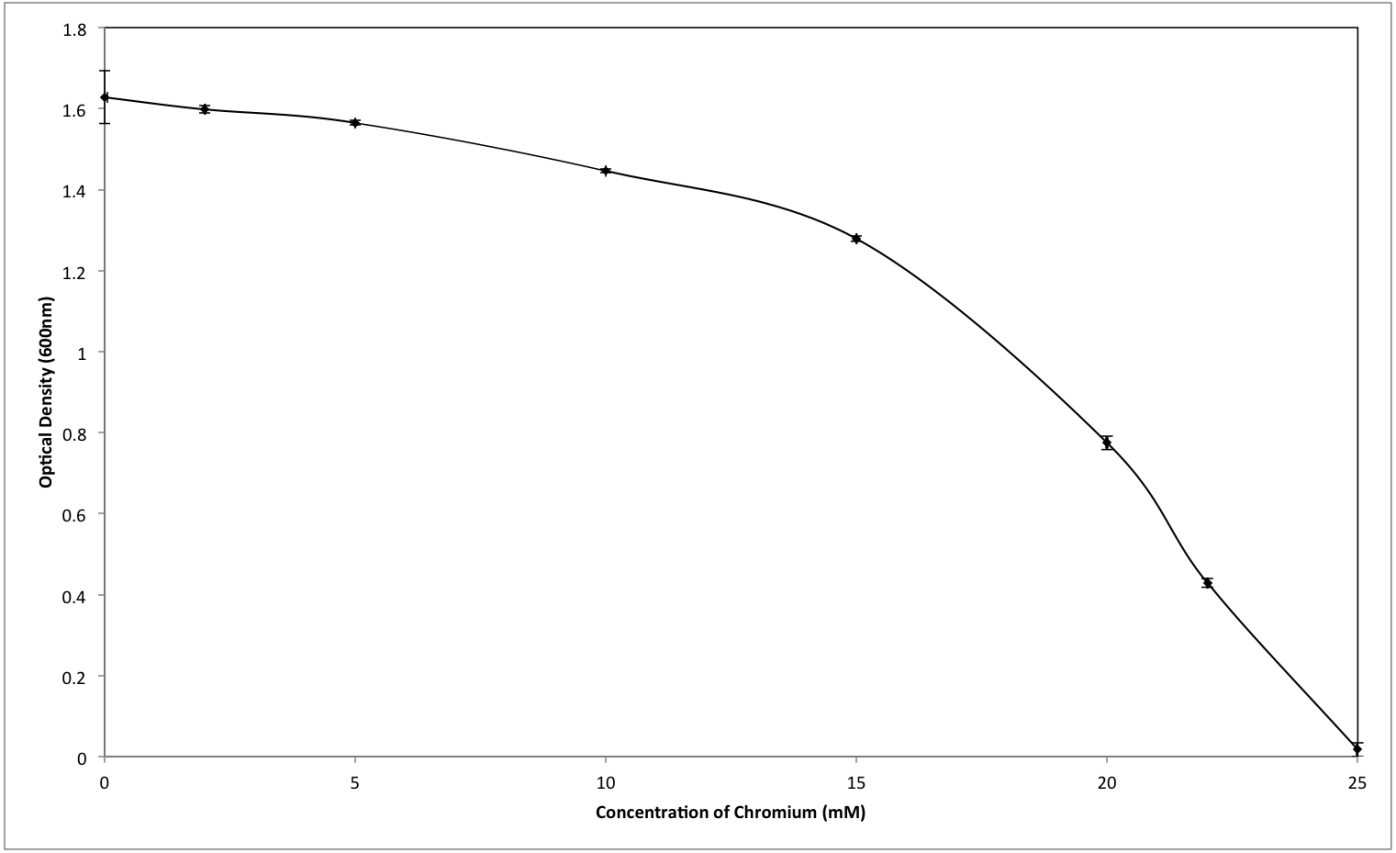
Effect of different concentrations of hexavalent chromium on the growth of *Staphylococcus aureus* strain K1. Each data point indicates the average performance of 03 runs.

Although the growth of bacterium K1 seemed to be uninhibited by presence of chromium, but it exhibited much slower growth rate in the presence of metal (Cr^6+^) as reported in Fig. 3. Additionally, in the absence of metal (Cr^6+^), exponential growth of bacterium lasted for about 8 h whereas no such growth was observed when chromium was present in culture medium (Fig. 3). Furthermore, *Staphylococcus aureus* K1 showed visible growth in Tryptic Soy Broth (TSB) medium containing chromium (hexavalent) with no significant inhibition (although growth rate was reduced) in bacterial growth up to maximum 22 mM Cr^6+^ as depicted in Fig. 4. Moreover, minimum inhibitory concentration of other metals such as lead, copper, zinc, manganese, cobalt, mercury and silver against *S. aureus* K1 was in following order i.e., Co^2+^: 18 mM; Zn^2+^: 15 mM; Pb^2+^: 10 mM; Mn^2+^: 8 mM; Ag^2+^: 5 mM; Cu^2+^: 3 mM; Hg^2+^: 1 mM.

### Bacterial antibiotic resistance

The bacterium *Staphylococcus aureus* K1 was tested against eighteen antibiotics. It was observed that the isolate K1 was sensitive to ceftriaxone, ampicillin, amoxicillin, imipenem, cefoxitin, ciprofloxacin, gentamycin, vancomycin, ertrapenem, amikacin, piperacillin/tazobactam, cefepime and intermediate sensitive to sulphamethoxazole/trimethoprim, meropenem and ofloxacin as mentioned in CLSI standards and was resistant to ceftazidime, aztreonam and nalidixic acid (Table 2).

**Table 2:** Antibiotic sensitivity pattern of chromium tolerant *Staphylococcus aureus* strain K1. Each data point indicates the average performance of 3 runs.

Sr. #	Antibiotic	Code	Concentration (µg/disc)	Sensitive/ Resistant	Diameter of zone of inhibition (mm)	Standards of diameter of zone (mm) of inhibition (CLSI)		
						Resistant	Intermediate	Sensitive
1	Ceftriaxone	CRO	30	Sensitive	22.00	≤13	14–20	≥21
2	Ampicillin	AMP	10	Sensitive	32.60	≤28	–	≥29
3	Amoxycillin	AMC	30	Sensitive	21.50	≤19	–	≥20
4	Meropenem	MEM	10	Sensitive	17.25	≤15	16–18	≥19
5	Imipenem	IPM	10	Sensitive	18.74	≤13	14–15	≥16
6	Sulphamethoxazole/ Trimethoprim	SXT	25	Sensitive	12.14	≤10	11–15	≥16
7	Nalidixic acid	NA	30	Resistant	–	≤13	14–18	≥19
8	Cefoxitin	FOX	30	Sensitive	18.10	≤14	15–16	≥18
9	Ciprofloxacin	CIP	5	Sensitive	21.30	≤15	16–20	≥21
10	Ofloxacin	OFX	5	Sensitive	15.80	≤12	13–15	≥16
11	Ceftazidime	CAZ	30	Resistant	–	≤14	15–17	≥18
12	Aztreonam	ATM	30	Resistant	–	≤15	16–21	≥22
13	Gentamycin	CN	10	Sensitive	16.13	≤12	13–14	≥15
14	Vancomycin	VA	30	Sensitive	18.35	–	–	–
15	Ertrapenem	ETP	10	Sensitive	20.17	–	–	–
16	Amikacin	AK	30	Sensitive	19.80	≤14	15–16	≥17
17	Piperacillin/ Tazobactam	TZP	110	Sensitive	21.00	≤19	–	≥20
18	Cefepime	FEP	30	Sensitive	20.50	≤14	15–17	≥18

### Effect of contact time (hours) on reduction of chromium (Cr^6+^) by *Staphylococcus aureus* strain K1

**Figure 5:**
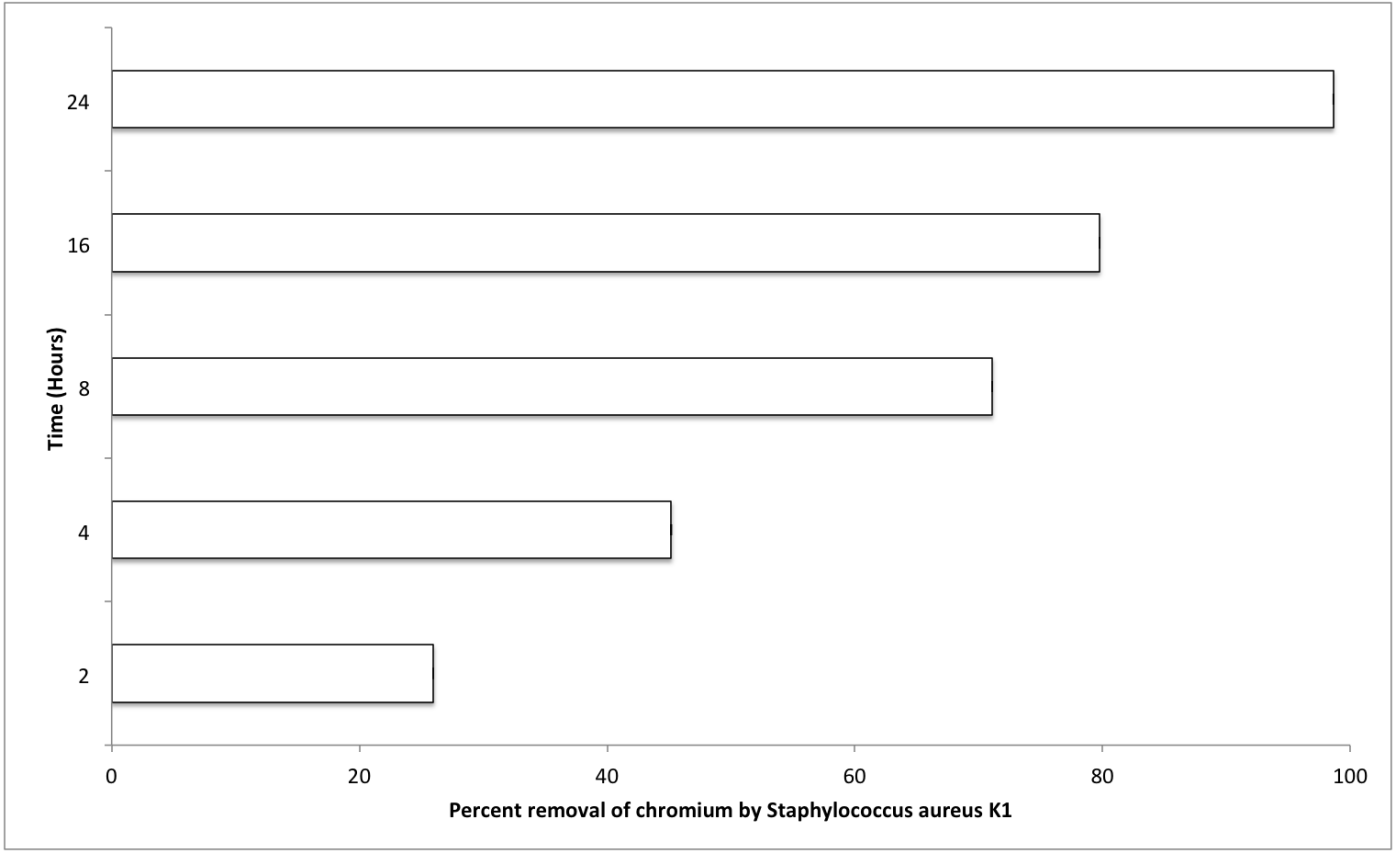
Effect of contact time (hours) on chromium removal capacity of *Staphylococcus aureus* K1.

The most important factors that can affect the various physiological processes of growing bacterium include temperature, pH, and salt concentration (0.5% NaCl). Optimum values for these factors were also evaluated for *S. aureus* strain K1. It was observed that *S. aureus* strain K1 exhibited maximum growth at 35 °C and pH 8. Under optimum growth conditions, biosorption (metal removal) capacity of strain K1 in the presence of chromium (hexavalent) increased with increasing the contact period (Fig. 5). It was observed that *Staphylococcus aureus* K1 removed about 26%, 45%, 71%, 80% and 99% Cr^6+^ (1 mM) from the medium after 2, 4, 8, 16 and 24 h, respectively (Fig. 5).

### FTIR analysis of bacterium *S. aureus* strain K1

**Figure 6:**
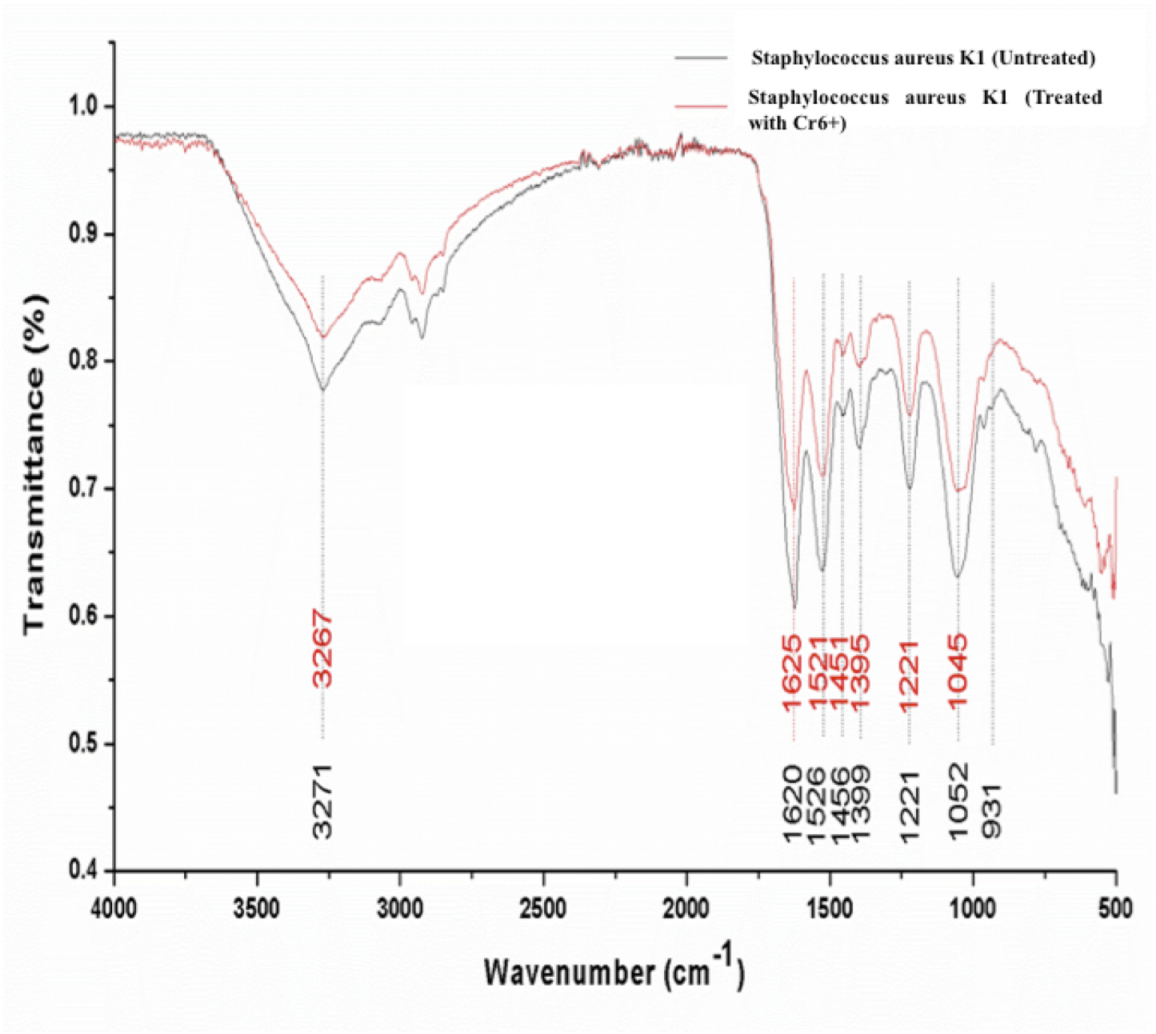
FTIR spectrum of *Staphylococcus aureus* strain K1 cells in the absence (untreated) and presence (treated) of Cr^6+^ (1 mM).

Significant differences were observed in the overall infrared spectrum of cells with and without chromium treatment. IR spectrum of *S. aureus* K1 cells with chromium (1 mM) and without chromium were studied in the range of 500–4,000 cm^−1^ that reflected the different absorption and stretching peaks validating the complex interactive nature of cellular surface (Fig. 6). In addition, the functional groups, involved in chromium sorption, were ionizable including carboxyl, hydroxyl groups and amino groups (Fig. 6).

## Discussion

From the results, it was evident that metal tolerant bacteria can be isolated from metal polluted sites. As in this study, we have isolated and characterized an indigenous *Staphylococcus aureus* strain K1 from metal polluted environment that can tolerate up to 22 mM of Cr^6+^. Several studies had reported the similar results in which metal tolerant bacteria were isolated from metal contaminated habitats (Shakoori, Makhdoom & Haq, 2000; Camargo et al., 2003; Viti, Pace & Giovannetti, 2003; Thacker et al., 2006; Wang et al., 2010; Alam & Ahmad, 2011; Zhang et al., 2013; Khan et al., 2015; Oaikhena et al., 2016). Our results also corroborated the findings of Mustapha & Halimoon (2015) who isolated 21 bacteria from electroplating industry and reported only five of them to be chromium tolerant (up to 50 mg/L). However the growth rate of bacterial isolates in the presence of toxic metal was slow as already reported in various studies around the globe (Basu, Bhattacharya & Paul, 1997; Shakoori, Makhdoom & Haq, 2000; Zhang et al., 2013; Ahemad, 2014). The reason for much slower growth rate in our case could be attributed to elevated chromium concentrations (22 mM) whereas other researcher had used lower levels (2 mM) of Cr^6+^ (Zhang et al., 2013). Similarly, use of nutrient rich medium for bacterial growth could also undermine the toxic effects of metal(s) on bacterial growth. For example, Thacker & Madamwar (2005) evaluated the effect of various concentrations of metal on bacterial growth using nutrient rich culture media having tryptone and yeast extract. Under such conditions, chromium (Cr^6+^) could form complex with organic compounds (present in nutrient medium), which in result could undermine the toxic effects of chromium, becoming less effective against microbial metabolism (Caravelli, Giannuzzi & Zaritzky, 2008).

Although, degree of tolerance to different metals (Cr, Co, Zn, Pb, Mn, Ag, Cu, Hg) might vary according to bacterial genotype, type of metal; nature of metal and pH of culture media (Zhang et al., 2013). Such resistance to toxic metals might be attributed to other possible mechanisms such as ion exclusion, bioaccumulation of metal by microbes and production of low molecular weight binding proteins (Nies, 1999; Das, Dash & Chakraborty, 2016).

As far as resistance to antibiotics was concerned, it was found that the isolate K1 was sensitive to majority of antibiotics tested. These findings corroborated the results of other researchers who reported that non-pathogenic but metal tolerant bacterial isolates belonging to *Serratia* species were more susceptible to antibiotics (Ajithkumar, 2003; Jafarzade et al., 2012). Similarly, Shakoori et al. (2010) stated that arsenic-resistant *Bacillus anthracis and Citrobacter freundii* were found sensitive to tetracycline, kanamycin, erythromycin and nalidixic acid while *Klebsiella oxytoca* was found resistant to all of these antibiotics. Others observed that *Bacillus species* exhibited sensitivity to streptomycin, tetracycline, chloramphenicol, norfloxacin, neomycin, rifampicin, nalidixic acid and cotrimoxazole, while being resistant to kanamycin, ampicillin and methicillin (Samanta et al., 2017). These results were also in line with reports from Abakiliki (Ikeagwu, Amadi & Iroha, 2008) suggesting that *Staphylococcus aureus* was sensitive to ofloxacin and gentamicin. Although *Staphylococcus aureus* K1 was resistant to ceftazidime, aztreonam and nalidixic acid that could be related to occurrence of genes responsible for heavy metal and antibiotic resistance together (Silver, 1988).

*Staphylococcus aureus* strain K1 was an efficient reducer of hexavalent chromium as depicted in Fig. 5. The removal percentage of chromium (Cr^6+^) was much more higher than previously reported values for Cr^6+^ removal by other *Staphylococcus* species (Shakoori et al., 2010; Zhang et al., 2013) and other bacteria (Li & Krumholz, 2009). Microbial reduction of chromium ion can be either extracellular or intracellular (Campos, Martinez-Pacheco & Cervantes, 1995; Ackerley et al., 2004; Li & Krumholz, 2009) that could undergo through biotransformation (in which Cr^6+^ is reduced to Cr^3+^ through microbial enzymatic processes) and/or by biosorption (metallic ions get adsorbed on cellular surfaces) (Cheung & Gu, 2003; Desjardin et al., 2003). These processes can be employed not only for the physical removal of metal (chromium) as precipitate (Cr(OH)_3_) but also can be helpful for decreasing the toxic effects of such metallic ions (Cheung & Gu, 2003). Hence *Staphylococcus aureus* K1 was an excellent reducer of chromium ion, it could be a potential candidate for bioremediation of chromium in that area as it could reduce nearly all of chromium present in medium after 24 h.

This rapid reduction of hexavalent chromium could be ascribed to surface binding due to the presence of different functional groups on cell wall and interior penetration (bioaccumulation) as reported previously (Rehman, Shakoori & Shakoori, 2006; Khan et al., 2015; Bingöl, Özmal & Akın, 2017; Long et al., 2017; Zhenggang et al., 2018). It was observed that the peaks attributed for amide linkages appearing at 1,620 and 1,526 cm^−1^were shifted respectively to 1,625 and 1,521 cm^−1^ on adsorption of chromium (Mungasavalli, Viraraghavan & Jin, 2007; Park, Yun & Park, 2005). The suppression and shift in peak intensity ranging 1,000–1,320 cm^−1^, denoting the presence of phosphorous and carbon containing oxygen atoms, indicated their interaction with Cr^+6^ (Das & Guha, 2007). It seems that the bacterial cells have undergone the oxidation during biosorption of chromium that has resulted into changes in overall IR spectrum (Park, Yun & Park, 2005; Long et al., 2017). These functional groups have previously been reported to interact with metal ions (cations) (Bueno et al., 2008). In fact, metal ion could form coordination bonding with peptide bond with either of amino or carboxyl groups, or nitrogen and/ or oxygen (Pandi, Shashirekha & Swamy, 2009). In current study, shift in wavenumber from 3,271 to 3,267 cm^−1^ of secondary amide was because of stretching of N-H group in the secondary-amide proteins representing the participation of membrane proteins in chromium binding (Abhay, Rawat & Singh, 2016). Our results reaffirmed the findings of (Mungasavalli, Viraraghavan & Jin, 2007), who assumed that amino groups could have leading role in chromium binding with cell membrane, as they were main constituents of protein, carbohydrates and hexosamines present in cell membrane. Furthermore, similar altered FTIR spectra was also observed for chromium-treated cyanobacteria, suggesting that formation of chromium complex with membrane protein (Pandi, Shashirekha & Swamy, 2009). These changes in FTIR spectrum of *S*. *aureus* strain K1 were indicative of the metal bonding processes that had taken place on bacterial surface with different functional groups (Bueno et al., 2008).

## Conclusions

According to biochemical and molecular characterization, this bacterium belongs to genus *Staphylococcus* and sub-species *aureus* strain K1. It was able to grow in metal concentration up to 22 mM of Cr^6+^ and could remove 99% chromium (1 mM Cr^6+^) after 24 h under optimum growth conditions. On the basis of FTIR spectra, it can be deduced that carboxyl, amino and phosphate groups were involved in complexation with chromium. In addition, *Staphylococcus aureus* K1 was sensitive to different antibiotics and therefore can be used for bioremediation of contaminated wastewater and soils. Furthermore, this bacterium K1 could likely have competitive advantage over autochthonous or exogenous strains if utilized for bioremediation and could play a significant part in the bioremediation of chromium-contaminated environments.

##  Supplemental Information

10.7717/peerj.7726/supp-1 Data S1 Raw data

## Additional Information and Declarations

### DNA Deposition

The following information was supplied regarding the deposition of DNA sequences: The 16S r DNA gene sequence of *Staphylococcus aureus* strain K1 is available at GenBank: KX685332.

### Data Availability

The following information was supplied regarding data availability: The bacterium used in this study is deposited in 1st Fungal Culture Bank of Pakistan under accession no. FCBP-WB-729 in University of Punjab, Lahore Pakistan. The raw data is available as a Supplemental File.
